# Extending the PROMIS item bank “ability to participate in social roles and activities”: a psychometric evaluation using IRT

**DOI:** 10.1007/s11136-024-03666-4

**Published:** 2024-05-23

**Authors:** Guido L. Williams, Gerard Flens, Caroline B. Terwee, Edwin de Beurs, Philip Spinhoven, Muirne C. S. Paap

**Affiliations:** 1 LMcare, Zwolle, The Netherlands; 2 Institute of Psychology, Leiden University, Leiden, The Netherlands; 3 Quality Alliance Mental Health, Akwa GGZ, Utrecht, The Netherlands; 4 Department of Epidemiology and Data Science, Amsterdam UMC Location Vrije Universiteit, Amsterdam, The Netherlands; 5 Amsterdam Public Health Research Institute, Methodology, Amsterdam, The Netherlands; 6 Institute of Psychology, Leiden University, Leiden, The Netherlands; 7 Department of Research, Arkin GGZ, Amsterdam, The Netherlands; 8 Institute of Psychology, Leiden University, Leiden, The Netherlands; 9 Department of Psychiatry, Leiden University Medical Center, Leiden, The Netherlands; 10 Department of Child and Family Welfare, Faculty of Behavioural and Social Sciences, University of Groningen, Groningen, The Netherlands; 11 Department of Research and Innovation, Clinic Mental Health and Addiction, Oslo University Hospital, Oslo, Norway

**Keywords:** Psychometric properties, Assessment, PROMIS-APSRA, IRT, CAT

## Abstract

**Objective:**

Our objective was to explore whether the extension of the PROMIS item bank Ability to Participate in Social Roles and Activities (APSRA) with new items would result in more effective targeting (i.e., selecting items that are appropriate for each individual's trait level), and more reliable measurements across all latent trait levels.

**Methods:**

A sample of 1,022 Dutch adults completed all 35 items of the original item bank plus 17 new items (in Dutch). The new items presented in this publication have been translated provisionally from Dutch into English for presentation purposes. We evaluated the basic IRT assumptions unidimensionality, local independence, and monotonicity. Furthermore, we examined the item parameters, and assessed differential item functioning (DIF) for sex, education, region, age, and ethnicity. In addition, we compared the test information functions, item parameters, and θ scores, for the original and extended item bank in order to assess whether the measurement range had improved.

**Results:**

We found that the extended item bank was compatible with the basic IRT assumptions and showed good reliability. Moreover, the extended item bank improved the measurement in the lower trait range, which is important for reliably assessing functioning in clinical populations (i.e., persons reporting lower levels of participation).

**Conclusion:**

We extended the PROMIS-APSRA item bank and improved its psychometric quality. Our study contributes to PROMIS measurement innovation, which allows for the addition of new items to existing item banks, without changing the interpretation of the scores and while maintaining the comparability of the scores with other PROMIS instruments.

**Supplementary Information:**

The online version contains supplementary material available at 10.1007/s11136-024-03666-4.

## Plain English summary

The researchers wanted to develop a better questionnaire that asks how well people can participate in society and perform everyday activities. They added new questions to an existing questionnaire because they thought some important topics were missing and the questionnaire needed more questions for people who had more or less trouble doing things. They asked 1022 people from the Netherlands to answer 52 questions, and 17 of them were new. They used a mathematical model to see if the questions measured the same construct, and if they went from low levels to high levels of functioning. They also checked if people from different groups answered differently. The researchers found that the new questions were better at asking people who had trouble doing things, which is important for finding out if people have health problems. But one new question had issues with separating people who had different levels of trouble and might become outdated soon. This question should be tested more in people who have trouble doing things, like people who go to the doctor. In the end, the researchers said that they made the questionnaire better by adding new questions to the old ones, without changing what the score meant.

## Introduction

Participation in social roles and activities contributes strongly to good health throughout life [1–3] and could be considered one of the core outcomes of healthcare [4–8]. The ability to participate in social roles and activities (APSRA) reflects what is considered important for improving health and general wellbeing, besides the relief of symptom burden [4, 9, 10]. APSRA is also a key component of the ﻿International Classification of Functioning, Disability and Health (ICF), which is a universal conceptualization of health and disability by the ﻿World Health Organization (WHO) [11]. While the importance of APSRA seems clear, the definition and operationalization of this construct is complex. Therefore, it is important to develop valid and reliable measures of APSRA that capture its diversity and specificity across different groups and settings [12–14]. An important contribution toward this objective has been made by ﻿the Patient Reported Outcomes Measurement Information System (PROMIS). PROMIS aims to improve and harmonize the measurement of self-reported health outcomes by using Item Response Theory (IRT) [15–19] and applications such as Computerized Adaptive Tests (CAT) [20–29]). PROMIS has designed several IRT-based item banks, including the item bank APSRA for measuring participation that allows for more efficient and reliable measurement of this construct using CAT [30].

Overall, the psychometric properties of the item bank APSRA were reported as adequate, according to the PROMIS standards [23, 31]. However, a recent qualitative study suggested that the APSRA item bank could benefit from additional items to better capture the full breadth of the underlying construct for lower or higher functioning individuals [14]. Furthermore, it was suggested that the content validity could be improved, especially with regard to the ICF activity and participation subdomains acquisition of necessities (i.e., ﻿acquiring a place to live), education (i.e., gaining admission to school), managing finances (i.e., ﻿maintaining a bank account), community life (i.e., engaging in social or community associations), and religion and spirituality (i.e., engaging in religious or spiritual activities). Also, it was suggested that the item bank may lack a distinction between engagement in remunerative (i.e., compensated) and non-remunerative (i.e., uncompensated) employment, and domestic life activities. As a solution, van Leeuwen et al. [32] proposed to add 17 items to the PROMIS-APSRA item bank (see Table 2). These additional items were generated by means of a three-step approach: (1) Item generation for 16 ICF subdomains currently not covered by the item bank; (2) Evaluation of content validity through expert review and think-aloud interviews; and (3) Item revision in a consensus procedure, based on the results of step 2 [32]. Their research confirmed the relevance, comprehensibility, and comprehensiveness of the 17 proposed items. They recommended to further study the psychometric properties of these items using IRT analysis, and to see how this affects the decision to add these new questions to the current item bank.

The present study has two aims. First, we will evaluate whether the IRT assumptions of unidimensionality, local item independence, and monotonicity hold for the extended item bank; and whether the items are free from differential item functioning (DIF) and show adequate levels of fit of the IRT model used. Second, we will investigate whether adding the new items leads to more effective targeting, i.e., covers a broader and more representative spectrum of the latent trait. Ideally, the item bank would contain items that cover the entire range of latent trait values, so that all latent trait levels can be measured with adequate levels of reliability. Evidence of improved targeting would support the added value of the new items.

## Methods

### ﻿Participants

A sample of 1022 Dutch people was drawn from the general population, aged 18 years and older, by a certified data collection agency (DESAN Research Solutions). The net sample was representative of the adult Dutch population (maximum deviation of 2.5%) regarding age (young 18–39 years; middle 40–64 years; old 65 + years), sex, education (low, middle, high), region (north, east, south, west), and ethnicity (native, first-, and second- generation western immigrant, first- and second-generation non-western immigrant), when compared to reference data from Statistics Netherlands from 2019 [33] (Table 1).

**Table 1:** Demographics Dutch sample (N = 1022)

	n (%)
Education	
Primary	279 (27%)
Highschool/associate	403 (39%)
Bachelor/master	340 (33%)
Region	
North	126 (12%)
East	198 (19%)
South	218 (21%)
West	480 (47%)
Ethnicity mother	
Native	815 (80%)
Western immigrant	99 (9.7%)
Non-western immigrant	108 (11%)
Ethnicity father	
Native	818 (80%)
Western immigrant	97 (9.5%)
Non-western immigrant	107 (10%)
Ethnicity participant	
Native	779 (76%)
Western immigrant	126 (12%)
Non-western immigrant	117 (11%)
Sex	
Male	499 (49%)
Female	523 (51%)
Age, median (IQR)	49 (34, 63)

### Procedure

All participants were members of an existing internet panel. The internet panel consisted of members of the online PanelClix service that was commissioned by DESAN Research Solutions (a specialized Dutch agency for collecting, processing, and reporting data for market and opinion research) in order to put together the online panel. PanelClix issues points (Clix) which are managed and administered by EuroClix, who also ensures that PanelClix members can exchange their points for euros. The panel members received 100 points for participating in our research, worth approximately 1 euro. The participants received an invitation to voluntarily take part in an online survey through an internet browser. After being presented with an introductory text with a brief explanation of the purpose of the survey, participants were asked to provide information about their age, sex, education, zip code, and ethnicity. Next, the participants were asked to rate their general level of participation by answering the question *“How would you describe your ability to participate in social role activities?”* on a 4-point scale (1 = not limited, 2 = mildly limited, 3 = moderately limited, 4 = severely limited). Next, they were asked to complete all 52 items of the extended version of the PROMIS-APSRA item bank. The items were presented in the same order for all participants, starting with the 35 original items followed by the 17 new items. The items were displayed in blocks of 5 items. All items within a block had to be answered for the next block of items to be presented.

### Measures

The original PROMIS item bank consists of 35 negatively ﻿worded items (e.g., *“I have to limit social activities outside my home”*; item code SRPPER_CaPS1). The 17 new items were written in the same grammatical style as the original items (e.g., *“I have trouble acquiring my groceries”*; item code PEXP_2. See Table 2 for the complete list of items^1^). The items were scored on a 5-point Likert scale (5 = never, 4 = rarely, 3 = sometimes, 2 = usually, 1 = always), with higher scores indicating greater ability to participate (i.e., fewer limitations). The item bank does not specify a time frame (e.g., *“Think back over the past 30 days”*).

**Table 2:** Discrimination and threshold parameter estimates for the extended patient-reported outcomes measurement information system item bank for the ability to participate in social roles and activities 2.0

Code	Item	*a*	*b*1	*b*2	*b*3	*b*4
RP1	I have trouble doing my regular daily work around the house	Fixed	Fixed	Fixed	Fixed	Fixed
RP6	I have trouble meeting the needs of my friends	Fixed	Fixed	Fixed	Fixed	Fixed
SRPPER_CaPS1	I have to limit social activities at home	Fixed	Fixed	Fixed	Fixed	Fixed
SRPPER01r1	I have trouble meeting the needs of my family	Fixed	Fixed	Fixed	Fixed	Fixed
SRPPER02r1	I am limited in doing my work (include work at home)	Fixed	Fixed	Fixed	Fixed	Fixed
SRPPER03r1	I have to limit social activities outside my home	Fixed	Fixed	Fixed	Fixed	Fixed
SRPPER04_CaPS	I have trouble participating in recreational activities with others	Fixed	Fixed	Fixed	Fixed	Fixed
SRPPER05_CaPS	I have trouble doing everything for my family that I feel I should do	Fixed	Fixed	Fixed	Fixed	Fixed
SRPPER06_CaPS	I have trouble accomplishing my usual work (include work at home)	Fixed	Fixed	Fixed	Fixed	Fixed
SRPPER07_CaPS	I have trouble doing all of the family activities that I feel I should do	Fixed	Fixed	Fixed	Fixed	Fixed
SRPPER08_CaPS	I have trouble doing all of the family activities that are really important to me	Fixed	Fixed	Fixed	Fixed	Fixed
SRPPER09_CaPS	I have trouble doing everything for work that I want to do (include work at home)	Fixed	Fixed	Fixed	Fixed	Fixed
SRPPER11_CaPS	I have trouble doing all of my regular leisure activities with others	Fixed	Fixed	Fixed	Fixed	Fixed
SRPPER13_CaPS	I have to limit social activities with groups of people	Fixed	Fixed	Fixed	Fixed	Fixed
SRPPER14r1	I have to limit my regular family activities	Fixed	Fixed	Fixed	Fixed	Fixed
SRPPER15_CaPS	I have to limit the things I do for fun with others	Fixed	Fixed	Fixed	Fixed	Fixed
SRPPER16r1	I have to do my work for shorter periods of time than usual (include work at home)	Fixed	Fixed	Fixed	Fixed	Fixed
SRPPER17r1	I feel limited in the amount of time I have for my family	Fixed	Fixed	Fixed	Fixed	Fixed
SRPPER18_CaPS	I have trouble doing all of the family activities that I want to do	Fixed	Fixed	Fixed	Fixed	Fixed
SRPPER20_CaPS	I have trouble doing all of the activities with friends that are really important to me	Fixed	Fixed	Fixed	Fixed	Fixed
SRPPER21_CaPS	I have trouble doing all the leisure activities with others that I want to do	Fixed	Fixed	Fixed	Fixed	Fixed
SRPPER22_CaPS	I have trouble keeping up with my family responsibilities	Fixed	Fixed	Fixed	Fixed	Fixed
SRPPER23_CaPS	I have trouble doing all my usual work (include work at home)	Fixed	Fixed	Fixed	Fixed	Fixed
SRPPER26_CaPS	I have trouble doing all of the work that is really important to me (include work at home)	Fixed	Fixed	Fixed	Fixed	Fixed
SRPPER28r1	I have to limit my regular activities with friends	Fixed	Fixed	Fixed	Fixed	Fixed
SRPPER31_CaPS	I have trouble taking care of my regular personal responsibilities	Fixed	Fixed	Fixed	Fixed	Fixed
SRPPER35_CaPS	I have trouble doing everything for my friends that I want to do	Fixed	Fixed	Fixed	Fixed	Fixed
SRPPER36_CaPS	I have trouble doing all of the activities with friends that I feel I should do	Fixed	Fixed	Fixed	Fixed	Fixed
SRPPER37_CaPS	I have trouble doing all of the work that I feel I should do (include work at home)	Fixed	Fixed	Fixed	Fixed	Fixed
SRPPER42r1	I feel limited in my ability to visit friends	Fixed	Fixed	Fixed	Fixed	Fixed
SRPPER43r1	I have trouble keeping in touch with others	Fixed	Fixed	Fixed	Fixed	Fixed
SRPPER46_CaPS	I have trouble doing all of the activities with friends that I want to do	Fixed	Fixed	Fixed	Fixed	Fixed
SRPPER47_CaPS	I have trouble keeping up with my work responsibilities (include work at home)	Fixed	Fixed	Fixed	Fixed	Fixed
SRPPER54_CaPS	I have trouble doing everything for my friends that I feel I should do	Fixed	Fixed	Fixed	Fixed	Fixed
SRPPER55r1	I feel limited in the amount of time I have to visit friends	Fixed	Fixed	Fixed	Fixed	Fixed
PEXP_1	I have trouble doing what is needed to acquire a place to live	1.18	−3.34	−2.41	−1.29	−0.50
PEXP_2	I have trouble acquiring my groceries	2.45	−2.26	−1.50	−0.71	0.06
PEXP_3	I have trouble taking care of my household	2.79	−1.84	−1.08	−0.22	0.49
PEXP_4	I have trouble taking care of my loved ones, including animals	3.04	−2.03	−1.41	−0.58	0.18
PEXP_5	I have trouble engaging with strangers	2.00	−2.32	−1.44	−0.51	0.36
PEXP_6	I have trouble creating and maintaining formal relationships, such as with my employers, or (voluntary)organization	2.02	−2.20	−1.40	−0.52	0.32
PEXP_7	I have trouble creating and maintaining romantic relationships	1.69	−2.09	−1.25	−0.42	0.47
PEXP_8	I have trouble doing everything for my education/ training that I want to do	1.87	−2.07	−1.32	−0.36	0.33
PEXP_9	I am limited in doing my paid work or internship	2.33	−1.46	−1.06	−0.46	0.17
PEXP_10	I am limited in doing unpaid work	2.17	−1.55	−0.93	−0.30	0.31
PEXP_11	I have trouble arranging online business, such as making payments	1.20	−3.72	−2.77	−1.59	−0.58
PEXP_12	I have trouble controlling my finances (administer bank account)	1.28	−3.82	−2.61	−1.46	−0.48
PEXP_13	I have trouble doing community activities such as in a social association	2.73	−1.63	−0.96	−0.28	0.40
PEXP_14	I feel limited in the extent to which I can be socially and politically involved	1.93	−2.12	−1.29	−0.40	0.41
PEXP_15	I have trouble traveling, for example going on vacation or business trip	2.15	−1.76	−1.16	−0.43	0.22
PEXP_16	I have trouble using digital and social media, such as WhatsApp, email, Facebook	0.97	−4.20	−3.02	−1.73	−0.50
PEXP_17	I have trouble dividing my time between my family, work, friends, leisure time and myself	1.80	−2.20	−1.38	−0.30	0.55

Note: PEXP items are the new Dutch items with a first provisional, unofficial English translation(original item parameters are fixed); N = 1022; discrimination = a, treshold = b

### Psychometric analysis

All analyses were performed in R version 4.1.2 [34]. The main packages used for the IRT analysis were mirt version 1.36.1 [35], mokken version 3.0.6 [36, 37], and lordif version 0.3–3 [38].

### IRT assumptions

In order to evaluate the incremental value of the new items, we first evaluated whether the items in the extended item bank adhered to the assumptions underlying most IRT models: unidimensionality, local item independence, and monotonicity; and whether the items were DIF-free [17, 18, 39, 40].

#### Dimensionality

Unidimensionality is a key assumption of the most frequently used IRT models. It means that the responses to a set of items can be sufficiently explained by a single latent trait, and it allows for the estimation of item parameters and latent person scores on a common scale. In keeping with the IRT framework employed throughout our psychometric analyses, an exploratory Mokken scale analysis (a nonparametric IRT based scaling technique) was performed to assess unidimensionality [41]. More specifically, the Automated Item Selection Procedure (AISP) was used. This procedure groups items into scales in an iterative manner. One of the aims in this procedure is to maximize the scalability coefficient H, which can be seen as an item-total correlation that has been corrected for the influence of item difficulty, i.e., item location. A Mokken scale is considered strong if H equals 0.5 or higher, moderate if 0.4 ≤ H < 0.5, and acceptable if 0.3 ≤ H < 0.4. Furthermore, the item scalability coefficients H_*j*_ should be ≥0.30 and the item-pair scalability coefficients H_*ij*_ should be positive [37]. As an additional check, we examined the model fit and the percentage of explained variance of the unidimensional graded response model (GRM; see section item calibration for more information). Following the recommendations of Maydeu-Olivares (2014) we used the M_2_ fit statistic in conjunction with the RMSEA_2_ and SRMR to assess adequate model fit (M_2_ *p* > 0.05; RMSEA_2_ < 0.089; SRMR < 0.05) [42].

#### Local item independence

Item pairs are locally independent when, controlling for the latent trait score, item responses show no association, i.e., the person parameter θ is not influenced by other factors than the trait level [16–18]. In order to test this assumption, we used Yen’s Q3 statistic with a residual correlation ≥ |0.2| as a critical value for signaling local item dependency [31, 43, 44].

#### Monotonicity

Monotonicity implies that when the latent trait level is increasing, so will the probability of endorsing a higher response category [19, 23]. We assessed monotonicity for the extended item bank by inspecting the category characteristic curves produced by confirmatory Mokken scale analysis. More specifically, the monotone homogeneity model (MHM) was estimated, which can be seen as a nonparametric counterpart of the GRM [36, 37, 45–47]. We evaluated the output for non-significant violations (#vi) and significant violations (#zsig). Additionally, violations of monotonicity were assessed by inspection of the critical values (CRIT) of the items. CRIT is a single statistic of several combined “goodness of fit” indicators [41] used in Mokken scaling. CRIT values should not exceed 80, while values below 40 are ideal, and values between 40 and 80 are considered acceptable violations [46, 48].

#### Differential item functioning

Differential item functioning (DIF) assesses the degree to which an item in a questionnaire functions differently for different groups. DIF occurs when two groups of respondents with similar ability levels but differing in some characteristic (such as sex, ethnicity, or age) have different probabilities of endorsing a response category on an item. DIF analysis is used to identify items that are biased in favor of one group to the detriment of another, thereby affecting the validity of the questionnaire. We investigated whether the items were sufficiently DIF-free with respect to age, sex, education, region, and ethnicity. We performed uniform and non-uniform DIF analyses for age (median split: ≤ 49 years, > 49 years), sex (male, female), education (low, middle, high), region (north, east, south, west), and ethnicity (native, western immigrant, non-western immigrant) [31].

With uniform DIF, the probability of endorsing an item will on average always be lower for one group, for all levels of θ. The two item characteristic curves for these groups would not intersect, i.e., would run more or less parallel to each other. Non-uniform DIF occurs when the probability of a response to an item depends both on the level of θ and the group membership of the respondent, resulting in intersecting item characteristic curves. DIF was evaluated by applying ordinal logistic regression models, using a McFadden’s pseudo *R*^*2*^ change of 2% as a criterion for DIF [38, 49], and by inspecting the item characteristic curves (ICCs) of items that were flagged for DIF.

### Item calibration

In order to assess the item parameters of the extended item bank, we used a GRM [50] where the item parameters for the original PROMIS items were set to the fixed US calibration values (as per PROMIS convention), and only those of the new items were estimated. The official PROMIS US item parameters were obtained via enquiry at HealthMeasures.^2^ The resulting estimated latent trait scores (i.e., θ) were scaled with a mean of 0 and a SD of 1, since this aligns best with keeping the scale as similar as possible to the original American PROMIS scale. Furthermore, a model with a freely estimated mean and SD showed negligible differences with a mean close to 0 and SD close to 1. Reliability was calculated for evaluating the quality of the test (i.e., scores are consistent and a good measure of the underlying trait). In order to examine item fit we calculated the generalized S-Χ^2^ statistic [51], which compares observed and expected response frequencies estimated by the IRT model, and quantifies differences between these frequencies. Items with a *p*-value smaller than 0.05 were considered indicative of poor fit.^3^ In addition, we assessed whether the discrimination parameters were sufficiently large (*a* > 1.0).

We used a Welch Two Sample t-test to test the difference between the item parameters of the new and old items, respectively. Effect sizes for the t-test were evaluated based on Cohen's (1988) recommendations [52]. Lastly, we visually examined the category response curves of the items, with the aim to gauge whether the item response categories were ordered as expected, and whether all item response categories had added value (i.e., were sufficiently non-overlapping). This provides an indication to what extent the response categories are able to differentiate between levels of functioning.

### Targeting

Targeting in IRT refers to the extent to which test items are appropriately matched to the latent trait level of the respondent. In order to achieve good targeting (i.e., to ensure accurate and meaningful measurements), it is important to use items that vary in location across the range of latent trait levels of the individuals completing the questionnaire. We evaluated the θ distribution of the extended item bank and examined whether the location (i.e., b_1_−b_4_) parameters of the new items covered a part of the latent trait range that had not yet been covered by the original items. For this we compared the test information functions and beta distributions for the original and extended item bank, in order to assess whether the new items broadened the range of θ  values that can be measured. Furthermore, we compared the absolute differences between the individual θ score estimated with the original item bank to those estimated with the extended item bank.

## Results

### Unidimensionality

The exploratory Mokken scale analysis indicated that the items in the extended item bank form a uniform scale. The total scale had an H-value of 0.56, which is indicative of a strong unidimensional scale with good item discriminatory power. All item scalability coefficients H_*j*_ exceeded 0.30 (range 0.31–0.65) and all the item-pair scalability coefficients H_*ij*_ were positive (see Table S1 in the online supplement). The proportion variance of 74% also supported unidimensionality. The overall fit of the model was unsatisfactory (M_2_(df = 1293) = 19,088.86, *p* < 0.001; RMSEA_2_ = 0.12; SRMSR = 0.14).

### Local item independence

Yen’s Q3 statistic flagged 34 ﻿item pairs for local item dependence. However, it should be noted that most violations were minor, only just exceeding the cut-off value of |0.2|. An exception was the residual correlation of 0.70 between item PEXP_12 (*“I have trouble keeping track of my finances (managing a bank account)”*) and PEXP_11 (*“I have trouble doing things online like making payments”*). Also, the residual correlations between item pair SRPPER23_CaPS (*“I have trouble doing all my usual work (include work at home)”*) and SRPPER37_CaPS (*“I have trouble doing all of the work that I feel I should do (include work at home)”*), and between item pair SRPPER35_CaPS (*“I have trouble doing everything for my friends that I want to do”*) and SRPPER36_CaPS (*“I have trouble doing all of the activities with friends that I feel I should do”*) were relatively high (respectively 0.48 and 0.41).

### ﻿Monotonicity

All items of the extended item bank had critical values below 40, and no violations of the assumption were observed when inspecting the monotonicity plots visually. Thus, we did not find evidence that this assumption was violated.

### Differential item functioning

﻿None of the items were flagged for DIF associated with sex, education, region, or ethnicity. For age, only item 17 was flagged for uniform DIF^4^ (SRPPER16r1 “*I have to do my work for shorter periods of time than usual (include work at home”)*). However, the degree of DIF is negligible (for more details, see supplemental material).

### Item calibration

The reliability for the extended item bank was high (0.98). The generalized S-Χ^2^ statistic showed that 23 of the 52 items (44%) had a *p*-value smaller than 0.05, possibly indicating a poor fit. Interestingly, this concerned 21 original items (40%) and only 2 new items (4%). The (freely) estimated discrimination and location (difficulty) parameter estimates for the new items are shown in Table 2.^5^

The discrimination parameter *a* ranged from 0.97 to 3.04 and from 1.99 to 4.88 for the new and old items respectively, indicating overall sufficient discriminating power. Only one item showed a value just below 1.00 (PEXP_16 *“I have trouble using digital and social media, such as WhatsApp, email, Facebook”*; *a* = 0.97). In general, the discrimination parameters of the new items were lower in comparison to the old (original) items. The Welch Two Sample t-test suggested that the *a* parameters of the new items were significantly lower than the old items, with a large effect size (mean *a* of new items = 1.98; mean *a* of old items = 3.92; difference = −1.94, 95% CI [−2.31, −1.58], *t*(33.79) = −10.83, *p* < 0.001; Cohen's *d* = −3.17, 95% CI [−4.10, −2.21]) (see Table 3).

**Table 3:** Item-fit statistics for the extended patient-reported outcomes measurement information system item bank for the ability to participate in social roles and activities 2.0

Item	Item code	S-X^2^	df	*p*-value
1	RP1	206.39	142	** < 0.001**
2	RP6	204.40	138	** < 0.001**
3	SRPPER_CaPS1	238.10	140	** < 0.001**
4	SRPPER01r1	181.96	145	**0.020**
5	SRPPER02r1	195.51	150	**0.007**
6	SRPPER03r1	189.32	151	**0.019**
7	SRPPER04_CaPS	250.96	157	** < 0.001**
8	SRPPER05_CaPS	183.14	132	**0.002**
9	SRPPER06_CaPS	161.41	137	0.076
10	SRPPER07_CaPS	145.30	126	0.115
11	SRPPER08_CaPS	141.24	124	0.138
12	SRPPER09_CaPS	177.60	149	0.055
13	SRPPER11_CaPS	178.38	122	**0.001**
14	SRPPER13_CaPS	177.74	145	**0.033**
15	SRPPER14r1	141.01	129	0.222
16	SRPPER15_CaPS	222.90	132	** < 0.001**
17	SRPPER16r1	247.32	183	**0.001**
18	SRPPER17r1	226.88	174	**0.004**
19	SRPPER18_CaPS	135.38	129	0.333
20	SRPPER20_CaPS	173.23	129	**0.006**
21	SRPPER21_CaPS	145.50	135	0.253
22	SRPPER22_CaPS	178.89	126	**0.001**
23	SRPPER23_CaPS	153.62	136	0.143
24	SRPPER26_CaPS	141.14	128	0.202
25	SRPPER28r1	143.04	130	0.205
26	SRPPER31_CaPS	158.47	132	0.058
27	SRPPER35_CaPS	129.79	130	0.498
28	SRPPER36_CaPS	189.87	130	** < 0.001**
29	SRPPER37_CaPS	183.10	141	**0.010**
30	SRPPER42r1	204.43	163	**0.015**
31	SRPPER43r1	248.80	191	**0.003**
32	SRPPER46_CaPS	172.63	129	**0.006**
33	SRPPER47_CaPS	148.87	139	0.268
34	SRPPER54_CaPS	185.44	135	**0.003**
35	SRPPER55r1	202.28	185	0.182
36	PEXP_1	228.38	193	**0.041**
37	PEXP_2	184.83	163	0.116
38	PEXP_3	172.31	175	0.543
39	PEXP_4	139.08	144	0.600
40	PEXP_5	197.05	184	0.242
41	PEXP_6	210.90	194	0.193
42	PEXP_7	259.91	240	0.180
43	PEXP_8	245.24	222	0.136
44	PEXP_9	242.69	225	0.199
45	PEXP_10	239.83	235	0.401
46	PEXP_11	197.63	182	0.203
47	PEXP_12	191.98	186	0.366
48	PEXP_13	194.70	187	0.335
49	PEXP_14	227.73	217	0.295
50	PEXP_15	260.99	223	**0.041**
51	PEXP_16	212.97	200	0.252
52	PEXP_17	234.39	217	0.199

*p* < 0.05 in bold

**Fig. 1:**
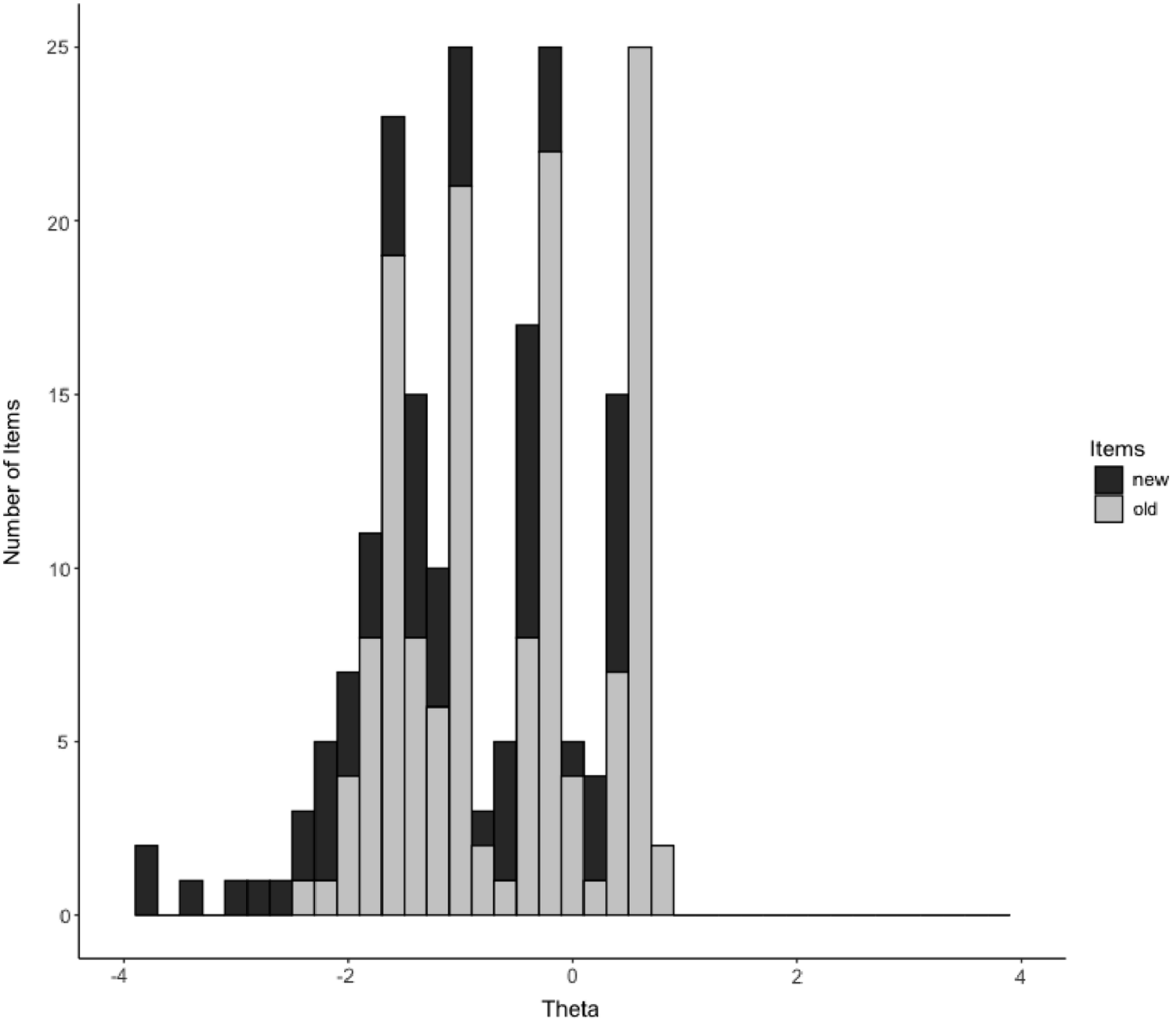
Stacked bar plot of location (b_1_, b_2_, b_3_, and b_4_) parameters

The location parameters (b_1_, b_2_, b_3_, and b_4_) ranged from −4.20 to 0.55 and from −2.49 to 0.73 for the new and old items, respectively. Figure 1 shows that targeting is improved substantially for the lower end of the scale by the new items (black bars) relative to the old items (grey bars). We refer to figure S2 in the online supplement for density plots of the item parameters grouped by old and new items.

The Welch Two Sample t-test indicated that the mean b parameters of the new items were significantly lower than the old items, with a medium effect size (mean beta of new items = −1.13; mean beta of old items = −0.62; difference = −0.51, 95% CI [−0.82, −0.20], *t*(107.18) = −3.26, *p* = 0.002; Cohen's *d* = −0.50, 95% CI [−0.81, −0.19]). These findings are consistent with a Two Sample t-test indicating that the raw scores for the new items were significantly higher with a small effect size (mean of new items = 4.01; mean of old items = 3.76; difference = 0.25, 95% CI [0.23, 0.27], *t*(53,142) = 24.37, *p* < 0.001; Cohen's *d* = 0.23, 95% CI [0.21, 0.24]). The mean raw score for all items (i.e., the extended item bank) was 3.84 (*sd *= 0.20; range: [3.61, 4.44]), and showed a left skewed distribution of -0.68 (*sd* = 0.31; range: [−1.73, −0.37]). The new items were more heavily (left) skewed than the old items (mean skewness new items = −0.98; *sd *= 0.38; range: [−1.73, −0.56]; mean skewness old items = −0.54; *sd* = 0.11; range: [−0.81, −0.37]).

**Fig. 2:**
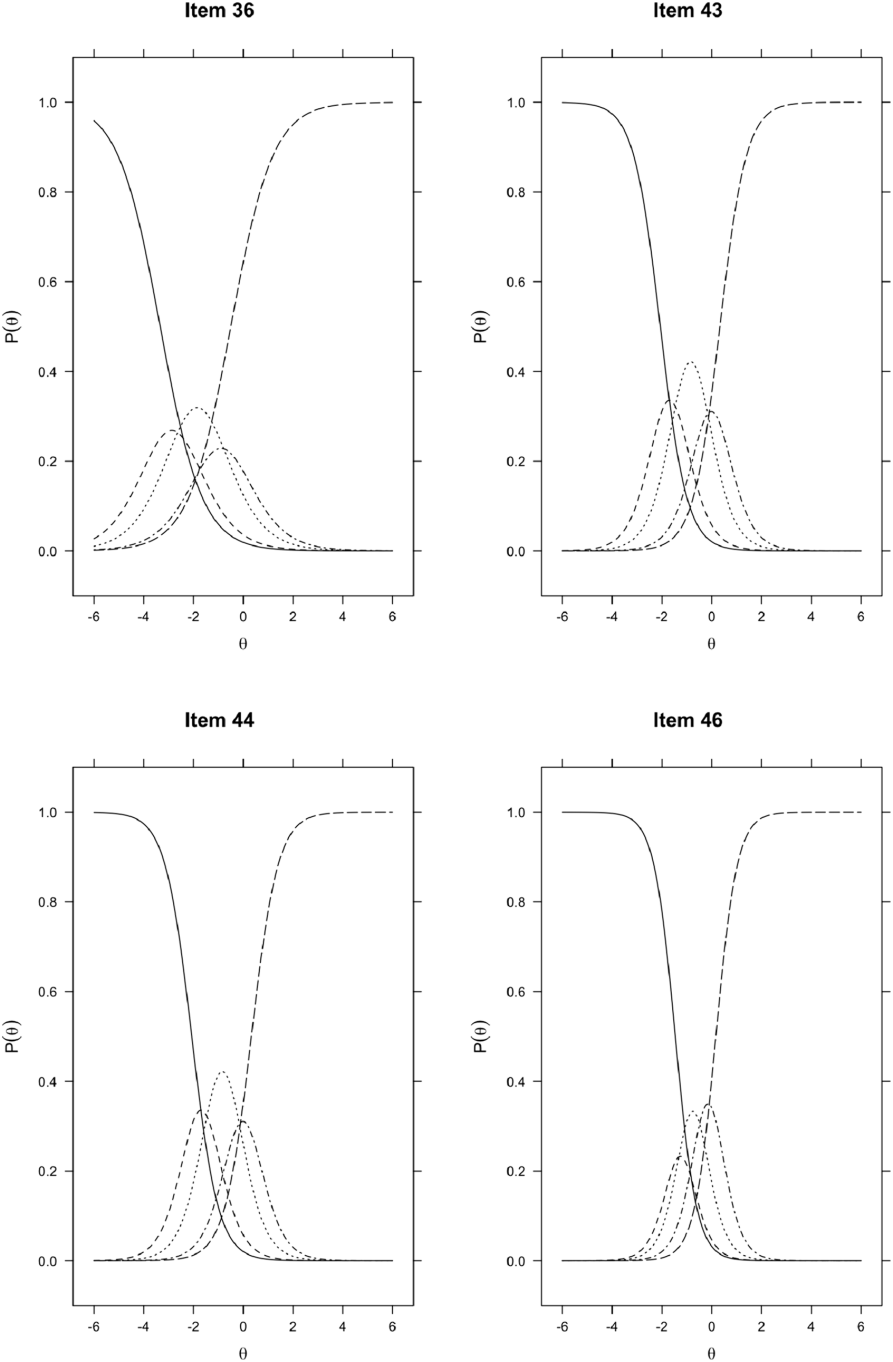

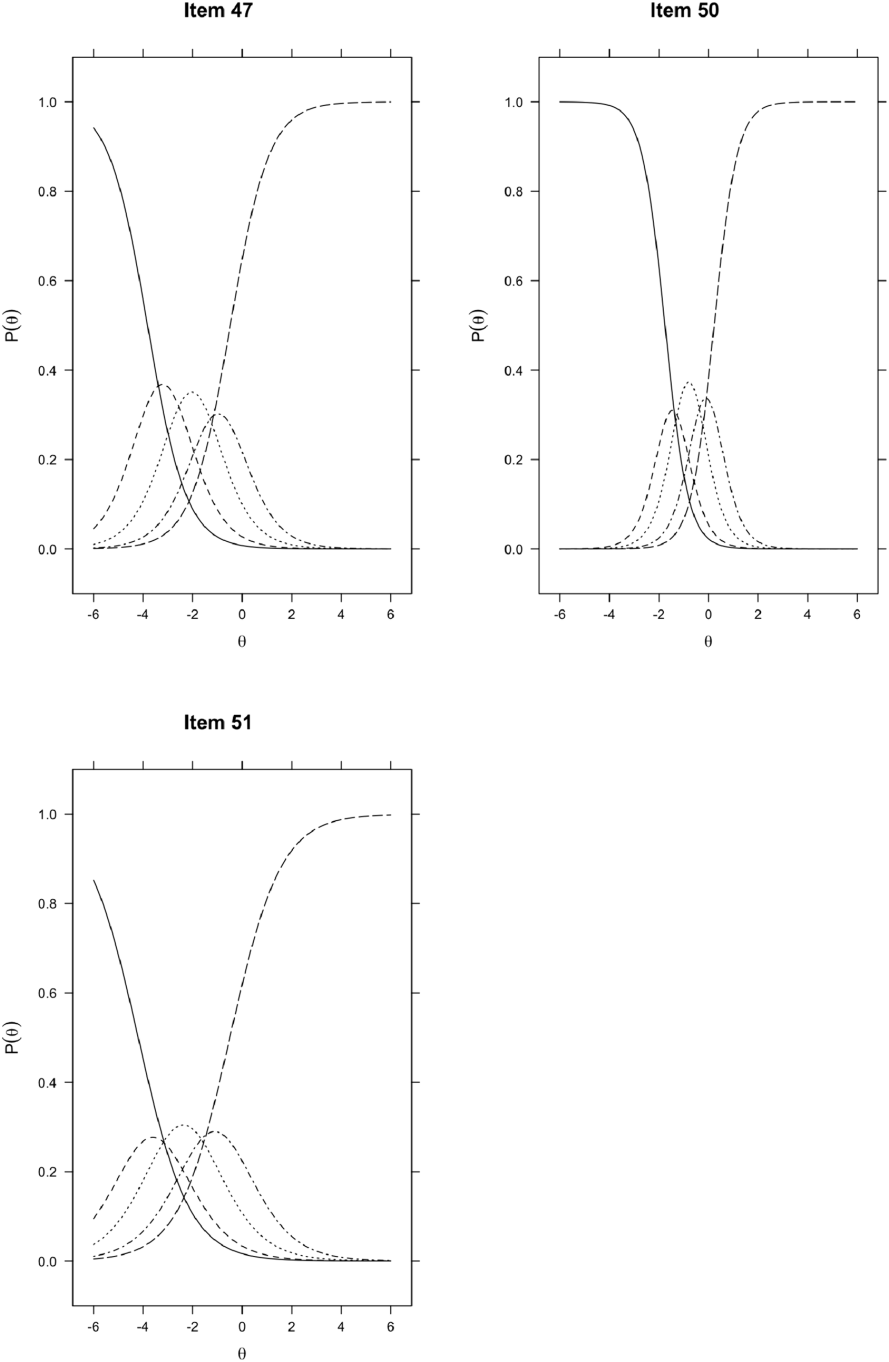
Item characteristic curves of items with less clear relations between θ and the probability of choosing a single response option

An examination of the trace lines of the probability functions from the extended item bank (i.e., the category response curves) showed that for some items of the extended item bank, it is less clear what response option (i.e., scoring category) is the most likely given a certain trait level (see Fig. 2). This seems true for item 36 (PEXP_1), item 43 (PEXP_8), item 44 (PEXP_9), item 46 (PEXP_11), item 47 (PEXP_12), item 50 (PEXP_15), and item 51 (PEXP_16).

In sum, the extended item bank showed high reliability, but many original items showed poor fit according to the generalized S-Χ^2^ statistic. Although, the new items had lower discrimination parameters, the lower location parameters of the new items showed that these items improved targeting people who reported low levels of social participation. Some items in the extended item bank had disordered response categories, meaning that the response option that was most likely for a given trait level was not always obvious.

### Targeting

**Fig. 3:**
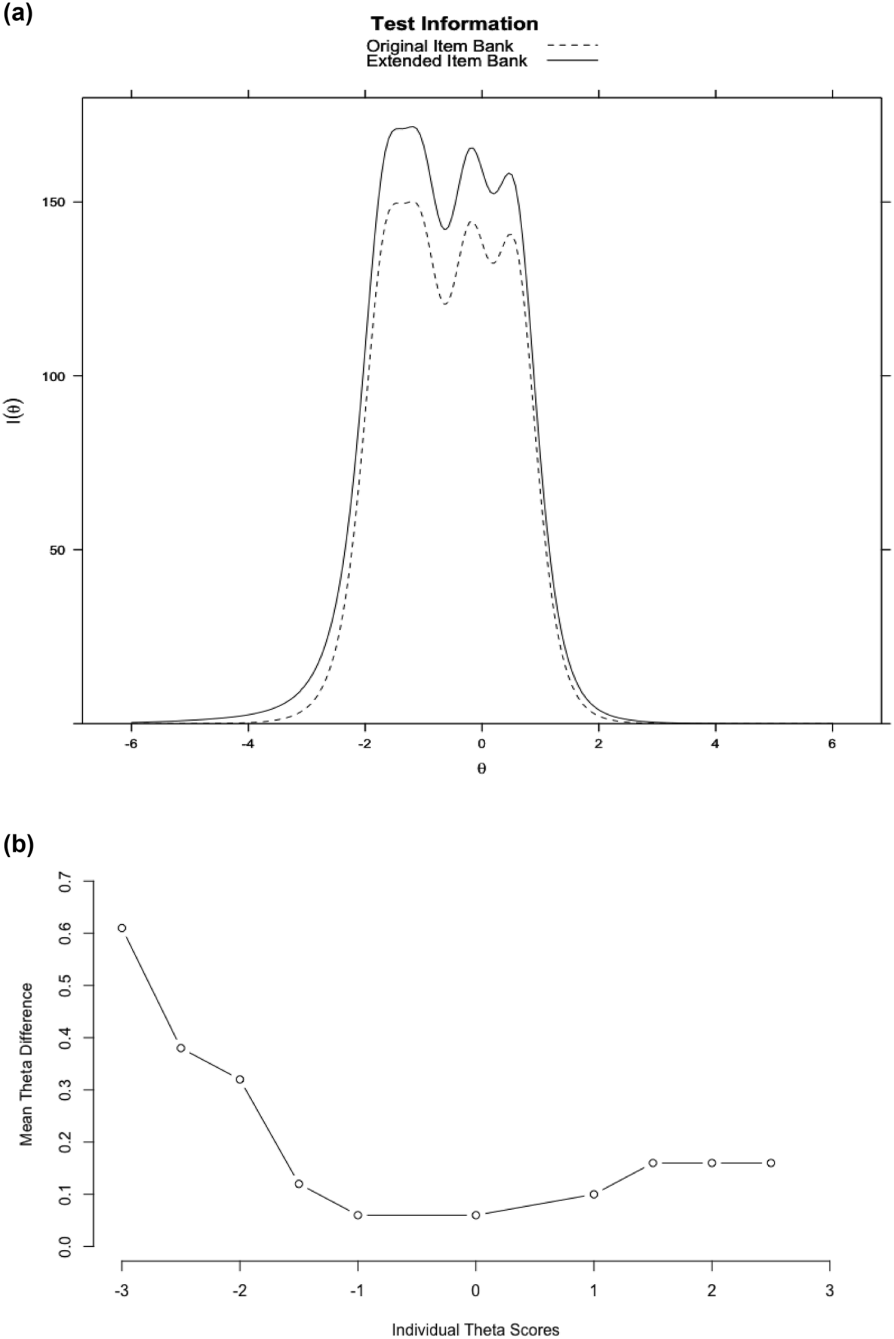
**a **Test information curves. **b** Absolute mean θ difference between item banks by θ score. Fig. 3b illustrates the absolute mean difference in individual θ scores between the original and extended item bank across various levels of θ. While the overall absolute mean difference for the entire group was 0.06, this discrepancy increased when comparing subgroups with different θ levels. For instance, when examining the absolute difference in individual θ scores between the item banks for subjects with an individual θ score of −2 or less, the absolute mean difference was 0.32. Notably, this effect is primarily observed at the lower end of the latent trait, i.e., in subjects with lower levels of participation. These findings indicate that the inclusion of new items in the extended item bank expands the measurement range, particularly at the lower end of the scale.

The test information function in Fig. 3a visualizes where the original and extended item banks are providing (the most) information relative to θ levels. It can be seen that the extended item bank covers a wider range of θ levels, especially, at the lower range (i.e., persons reporting lower levels of participation). This is consistent with our finding that the new items had significantly lower location parameters than the old items (see Fig. 1), meaning that they are possibly more suitable for measuring lower levels of participation.

The individual θ scores based on the original item bank ranged from −2.76 to 1.75, whereas the individual θ scores based on the extended item bank ranged from −3.11 to 1.91. A comparison of the individual θ scores from the original item bank (old items with fixed parameters) and the individual θ scores from extended item bank (old items with fixed parameters and new items with freely estimated parameters) showed a high correlation (r. = 0.99) and an absolute mean difference of 0.06 with an *sd* of 0.06. However, the absolute mean difference in θ scores between the original and extended item bank, was larger for individuals with lower θ scores (Fig 3b). This shows that the new items broaden the measurement range especially at the lower end of the scale.

## Discussion

This study applied IRT modeling to examine the psychometric properties of the extended PROMIS-APSRA item bank, including the basic IRT assumptions, differential item functioning, item fit and whether the new items improved the targeting of lower/higher levels of participation. Overall, we found sufficient support for the IRT assumptions, and we did not find substantial item bias in terms of DIF. The discrimination parameters of the new items were lower than those of the old items. However, the inclusion of the new items in the item bank enhanced the information function at the lower levels of participation, leading to better targeting of the lower range of the latent trait scale. Together, these findings suggest that extension of the PROMIS-APSRA item bank resulted in a meaningful improvement of the psychometric quality.

Although, many items seemed locally dependent, most violations were minor, and possibly an artefact of the fact that the items were displayed in blocks of 5 items at the same time [43]. An exception was the high residual correlation between the new items PEXP_12 (*“I have trouble keeping track of my finances (managing a bank account)”*) and PEXP_11 (*“I have trouble doing things online like making payments”*). This is likely due to the similarity in wording and content, making it harder for a respondent to distinguish the differences between these questions [23, 43, 44]. As a consequence, we advise against including both these items at the same time in a short form or CAT.

Our results indicated that item bias in terms of DIF was low. Only one item (item 17; SRPPER16r1 *“I have to do my work for shorter periods of time than usual (include work at home)”*) was flagged for uniform DIF due to age. The impact, however, seemed negligible, and we therefore suggest keeping this item in the item bank. We conclude that different subgroups with the same level of participation do not have different probabilities of endorsing an item response (i.e., the item parameters are invariant across different populations), and that the items are unbiased for all respondents, regardless of their sex, education, region, or ethnic background.

The generalized S-Χ^2^ statistic indicated that 21 items from the original item bank and 2 new items from the extended item bank had a potential misfit with the model. Item misfit occurs when an item does not conform to the expectations of the model, and the observed responses deviate significantly from the expected responses based on the model. Several factors can contribute to misfitting items, such as multidimensionality, guessing, local dependence, or cultural bias [51, 53, 54]. We ruled out multidimensionality and guessing as possible sources of misfit, since our analysis confirmed the unidimensional structure of the scale, and the items did not have correct or incorrect responses. However, we considered local dependence and cultural bias as plausible explanations. Local dependence occurs when the responses to two or more items are highly correlated, and the response to one item can predict the response to another item. This can lead to an overestimation of the test reliability and an underestimation of the standard errors of the item parameters. We detected some minor effects of local dependence in our data, but they were not sufficient to explain the misfit identified by the S-Χ^2^ statistic.

Since the misfit mainly occurred in the original items, and the parameters for the old items were fixed on US parameters while the new items were estimated based on Dutch data, cultural bias could be the most likely cause of the misfitting items in our scale. This might partly clarify why the model's overall fit was not satisfactory, even though the expanded item bank constitutes a robust unidimensional scale. The implications of these results warrant further investigation into the role of cultural factors in relation to item fitness, and we recommend retaining the items with statistical misfit in the extended item bank, for now.

The location parameters (b_1_, b_2_, b_3_, and b_4_) of the new items have significantly lower values than the old items. These findings suggest that the new items can be used to improve the measurement of the lower trait levels. Comparison of individual θ scores based on the original item bank and the extended item bank also support this conclusion.

The discrimination parameters (*a*) of the new items have significantly lower values, indicating that they are less able to differentiate between respondents with a high level of functioning and those with a low level of functioning compared to the old items. Nevertheless, the discrimination power for the new items is still sufficient. Only item PEXP_16 (*“I have trouble using digital and social media, such as Whatsapp, email, Facebook”*) showed a discriminating power just below 1 and a marginal Mokken scalability coefficient (H_*i*_ = 0.306). Therefore, item PEXP_16 is a serious candidate for exclusion from the item bank despite its low threshold values (starting at b1 ≈ −4.20), that could make this item eligible for measuring the latent trait score of respondents with severe impairments (i.e., in a clinical population, who are expected to generally have a lower ability to participate in social roles and activities). We suggest a critical study of this item in a clinical sample. We also advise to rephrase this item by removing specific examples (i.e. “I have trouble using digital and social media” or "I have trouble using digital and social media due to certain health-related challenges.") to prevent outdated wording in the future.

We also found that for 7 of the 17 new items, the category response curves were not peaked and adequately dispersed across all levels of the latent trait, making it is less clear what response option (i.e., scoring category) was the most likely given a certain θ value (see Fig. 2). This meant that not all response options contributed meaningfully to the estimation of trait levels. A visual inspection of the operating characteristic curves for these items suggests that using three rather than five response options may have been more appropriate for these items. However, we advise against using different numbers of response options for a subset of items, since it might be confusing for respondents to answer them.

### Strengths, limitations, and future research

This study has several strengths and limitations. One of the strengths is that we used a large [55] and representative, stratified, sample of the Dutch general population, which enhances the external validity and generalizability of the findings. Another strength is that this study built on a well-established item bank from a renowned system (PROMIS), and thus had a solid foundation for the development of a potentially more accurate measurement of participation in social roles and activities.

However, the study also has some limitations that may have affected the quality of the findings and the ability to answer the research questions. In order to ensure comparability with the original item bank, the parameters for the old items were fixed on US parameters, while the new items were estimated based on Dutch data. As argued by Terwee et al. [56], such an approach may have introduced some bias or inconsistency in the item calibration and scaling. Furthermore, it is crucial to recognize the intricacies associated with translating and culturally adapting new items. Notably, the newly proposed items were developed in Dutch, while the original items were developed in English. Therefore, further research is needed before the proposed items are incorporated into other language versions of the item bank. This study examined the psychometric properties in a non-clinical population. We strongly recommend that the item bank's applicability in clinical practice and for individuals with specific needs, such as those with low literacy, is examined in a future study. Moreover, this study did not test the predictive validity or responsiveness of the measure, which are important aspects for evaluating its usefulness in clinical practice and research. In order to address these important topics, we plan to conduct further studies, preferably by using CAT simulations, to examine the added value of the extended item bank in a clinical population, and to test its ability to detect changes over time and predict treatment outcomes.

## Conclusion

In conclusion, we found that the extended item bank showed good reliability and validity in the Dutch general population. Moreover, the extended item bank improved the measurement in the lower trait range, which is important for reliably assessing functioning in clinical populations. Our study also contributes to further innovation of PROMIS measurements, which allow for dynamic and flexible addition of new items to item banks, without changing the interpretation of the scores, and while maintaining the comparability of the scores with other PROMIS instruments. We hope that this study will stimulate further research on social participation and its measurement in different populations and contexts.

### Supplementary Information

Below is the link to the electronic supplementary material.

Supplementary material 1 (PDF 576.0 kb)

## Data Availability

The data that support the findings of this study are available upon reasonable request. Note that restrictions apply to the availability of these data, which were used under license for the current study, and for that reason are not publicly available.

## Footnotes

1 For the sake of readability, the new items presented in this publication have been provisionally translated from Dutch into English. If the new items are eligible to be included in the original English item bank, they need to undergo the standard PROMIS translation process for the English language, including back-translation and cognitive debriefing.

2 HealthMeasures is the dissemination and implementation hub for four state-of-the-science measurement systems: PROMIS®, NIH Toolbox®, Neuro-QoL™, and ASCQ-Me.®. Originally funded by the National Institutes of Health (NIH), it is now supported under a cost-recovery business model with no profit motivation at Northwestern University’s Feinberg School of Medicine. (https://www.healthmeasures.net).

3 Although it has been suggested that a p-value smaller than .05 may indicate poor fit [53, 57], there is still no consensus on the optimal cut-off value for the *p*-value. A Benjamini and Hochberg adjusted alpha level could be more suitable for controlling a false discovery rate [58, 59]. However, for individual testing, an alpha adjustment could be less appropriate [60]. ﻿An alpha adjustment might also emphasize the importance of statistical significance, whereas practical consequences should also be considered. After all, a statistical misfit does not automatically indicate that an item should be dropped, because it might still be clinically relevant; and at the same time keeping the item does not necessarily have a significant or practical impact on severity estimates [58]. After considering aforementioned points, we decided to use an alpha level of .05 without adjustment as significance threshold for the S-X^2^ *p*-value.

4 With uniform DIF, the probability of endorsing an item will on average always be lower for one group, for all levels of θ. The two item characteristic curves for these groups would not intersect, i.e., would run more or less parallel to each other.

5 The parameters of the original items are not presented because of property rights but can be made available upon request. To inquire about permissions, email help@healthmeasures.net.

## Code availability

The R codes used for analysis are available from the corresponding author.

## Declarations

### Software

All analyses were performed in R, version 4.1.2 [34]. The main packages used for the IRT analysis were mirt (version 1.36.1) [35],
