# Supplementary material for: Extending the PROMIS item bank “ability to participate in social roles and activities”: a psychometric evaluation using IRT
Source: Qual Life Res. 2024 May 23;33(8):2129–44. doi: 10.1007/s11136-024-03666-4 (PMC11286634; doi:10.1007/s11136-024-03666-4)
Supplement: Supplementary file 1 — Supplementary material 1 (PDF 576.0 kb) [file 11136_2024_3666_MOESM1_ESM.pdf]

SUPPLEMENT

Title: PROMIS Item Bank for Social Roles and Activities: A Psychometric Evaluation

Journal: Quality of Life Research

Authors: Guido L. Williams, Gerard Flens Caroline B. Terwee, Edwin de Beurs, Philip Spinhoven, Muirne C. S. Paap Corresponding author: Guido L. Williams, LMcare, Zwolle, The Netherlands; williamsgl@vuw.leidenuniv.nl; ORCID 0000-0001-8008-5750; Leiden University, Institute of Psychology, Leiden, The Netherlands

Figures S1a. – S1b.: Differential Item Functioning Analysis Plots

Figure S2. Density plots Item Parameters Grouped by Old and New Items

Figure S3. Bland-Altman plot Comparing Individual Theta Scores Between Old and New Item Bank

Table S1. Mokken Item Scalability Coefficients

Figure S1a. Density Plot of Trait Distributions by Age Group

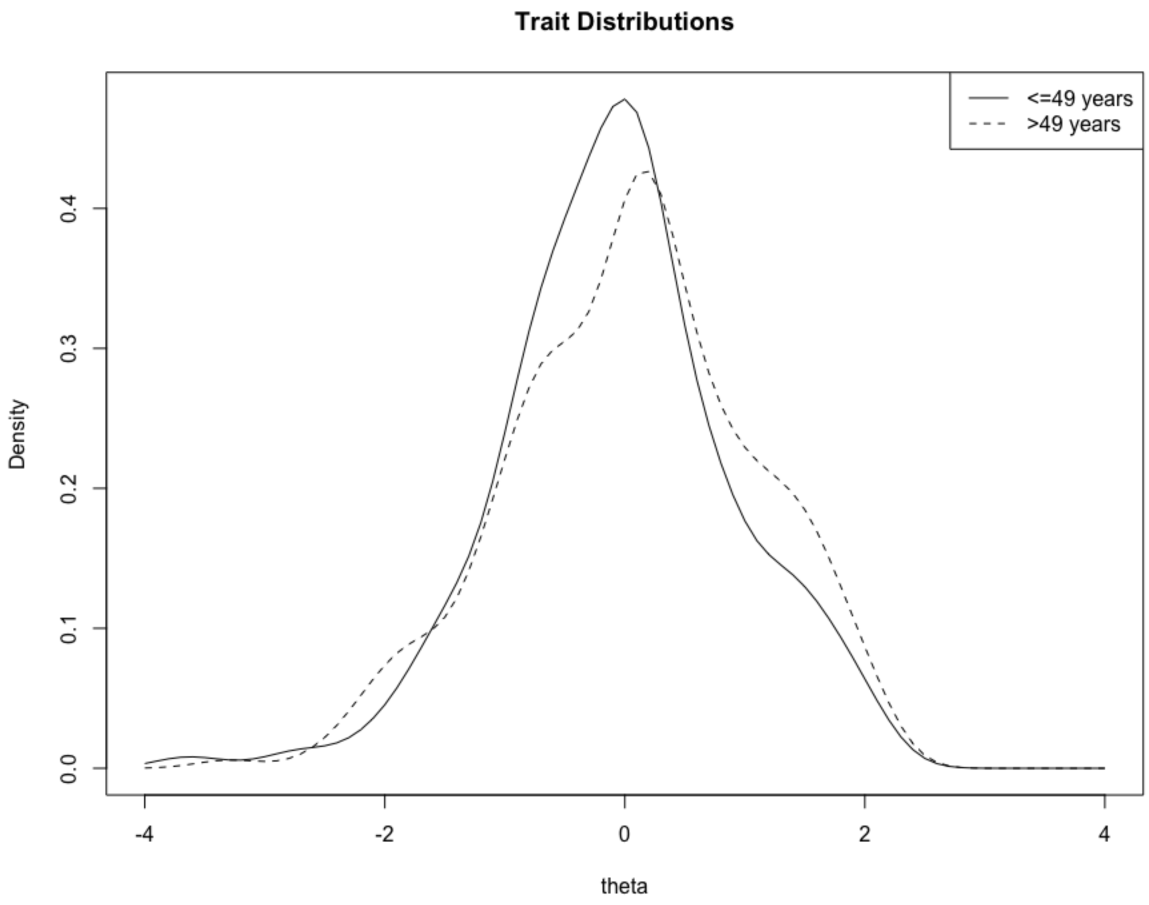

Note: This graph shows smoothed histograms of the ability (θ) to participate in social roles and activities levels for older (dashed line) and younger (solid line) participants. There is broad overlap in the distributions, where older individuals in general demonstrated a minor tendency for higher levels of participation than younger individuals.

Figure S1b. Operating Characteristic Curves of the Item *"I have to do my work for shorter periods of time than usual (include work at home),"* which shows uniform DIF with respect to age.

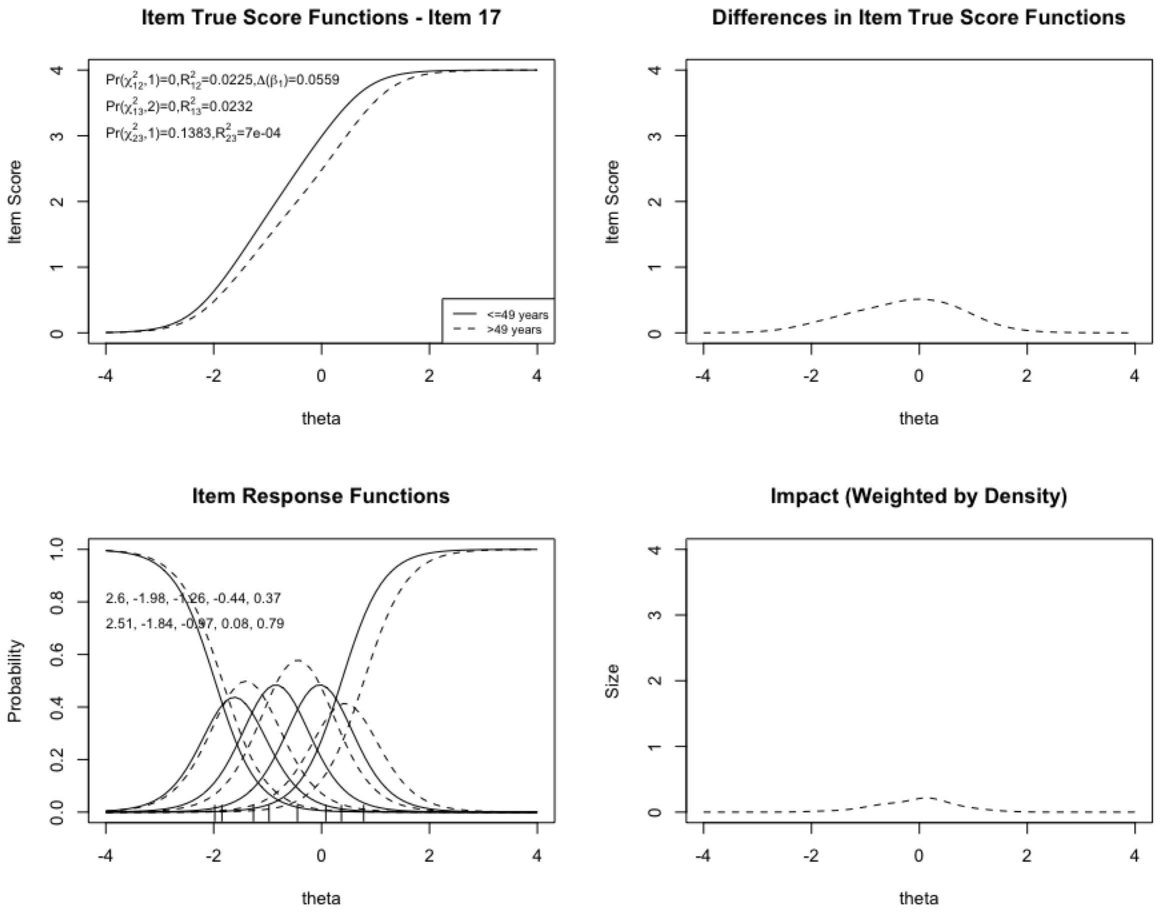

Note: The upper–left graph shows the item characteristic curves (ICCs) for the item for older (dashed curve) vs. younger (solid curve) participants. The two item characteristic curves for these groups do not intersect, indicating possible uniform DIF. The upper–right graph shows the absolute difference between the ICCs for the two groups, indicating that the difference is somewhat larger at the lower to average levels of participation (θ), but is still considered minimal. The lower–left graph shows the item response functions for the two groups, based on the slope and category threshold values by group. The lower–right graph shows the absolute difference between the ICCs (the upper– right graph) weighted by the score distribution for the focal group, i.e., older individuals (dashed curve in Figure 1), indicating minimal impact of age on the level of participation across θ.

Figure S1c. Impact of DIF items on Test Characteristic Curves.

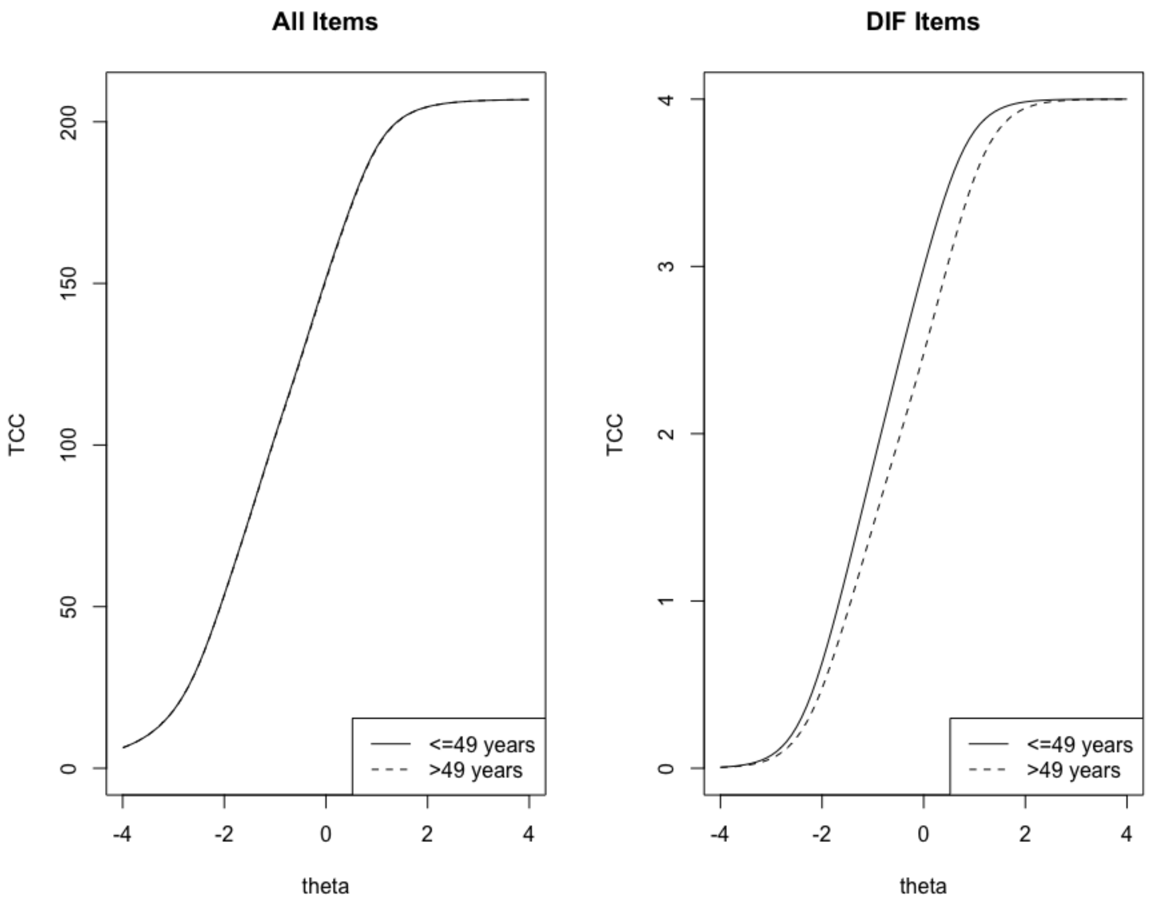

Note: These graphs show test characteristic curves (TCCs) for younger and older individuals using the age-group item parameter estimates. TCCs show the expected total scores for groups of items at each participation level (θ). The graph on the left shows these curves for all of the items (both items with and without DIF), while the graph on the right shows these curves for the subset of these items found to have DIF. These curves suggest that at the overall test level there is a difference in the total expected score at any participation level for older or younger individuals.

Figure S1d. Individual Score Differences between Scores Accounting and Not-Accounting for DIF.

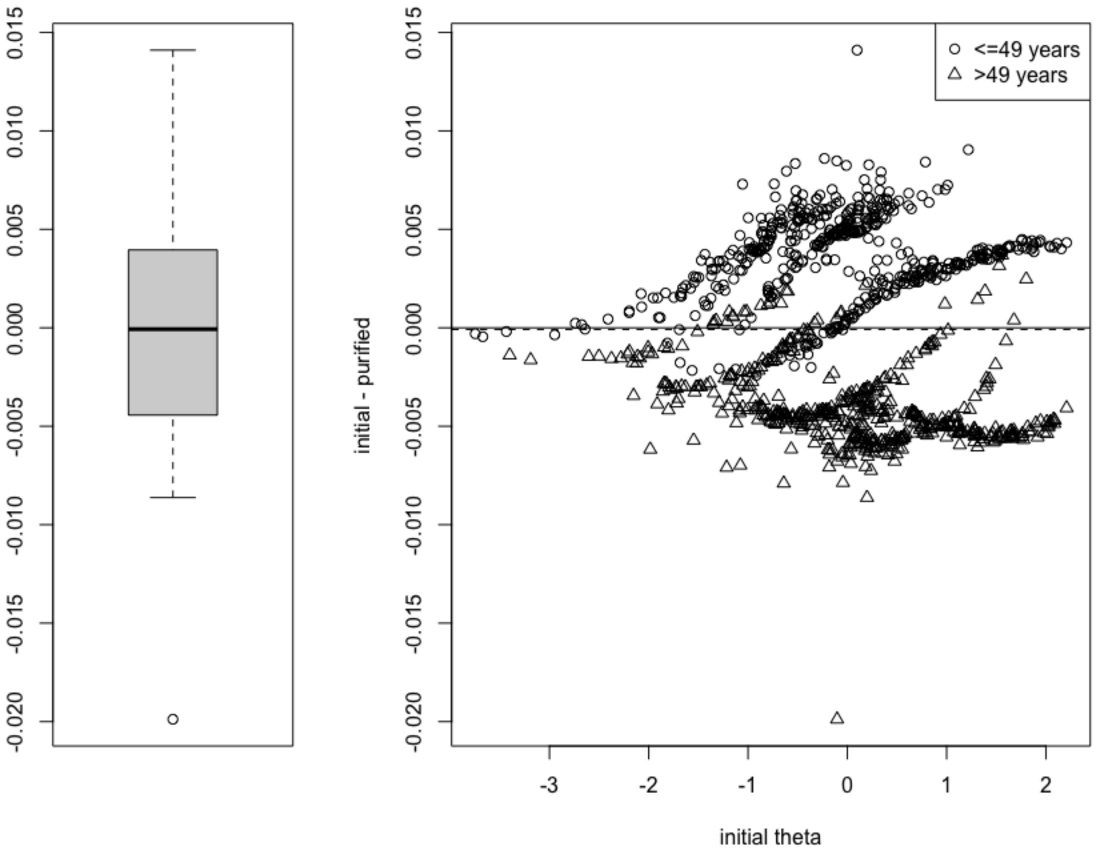

Note: These graphs show the difference in scores between using scores that ignore DIF and those that account for DIF. The graph on the left shows a box plot displaying these differences. The interquartile range, representing the middle 50% of the differences (bound between the bottom and top of the shaded box), range roughly from -0.004 to +0.004 with a median of approximately 0. In the graph on the right the same difference scores are plotted against the initial scores ignoring DIF ("initial θ"), separately for younger and older individuals. Guidelines are placed at 0.0 (solid line), i.e., no difference, and the mean of the differences (dotted line). Positive values on the y-axis indicate that the initial score (i.e., without accounting for DIF) minus the score accounting for DIF > 0, which means that the scores accounting for DIF must be lower than the initial score. Negative values on the y-axis indicate that the initial scores minus the scores accounting for DIF < 0, which means that the scores accounting for DIF must be higher than the initial scores. The older group shows negative values on the y-axis, indicating that when accounting for DIF, scores go up in this group. The younger

group shows positive values on the y-axis, indicating that when accounting for DIF, scores go down in this group.

Fig. S2. Density plots Item Parameters Grouped by Old and New Items

(b values are equal to theta levels)

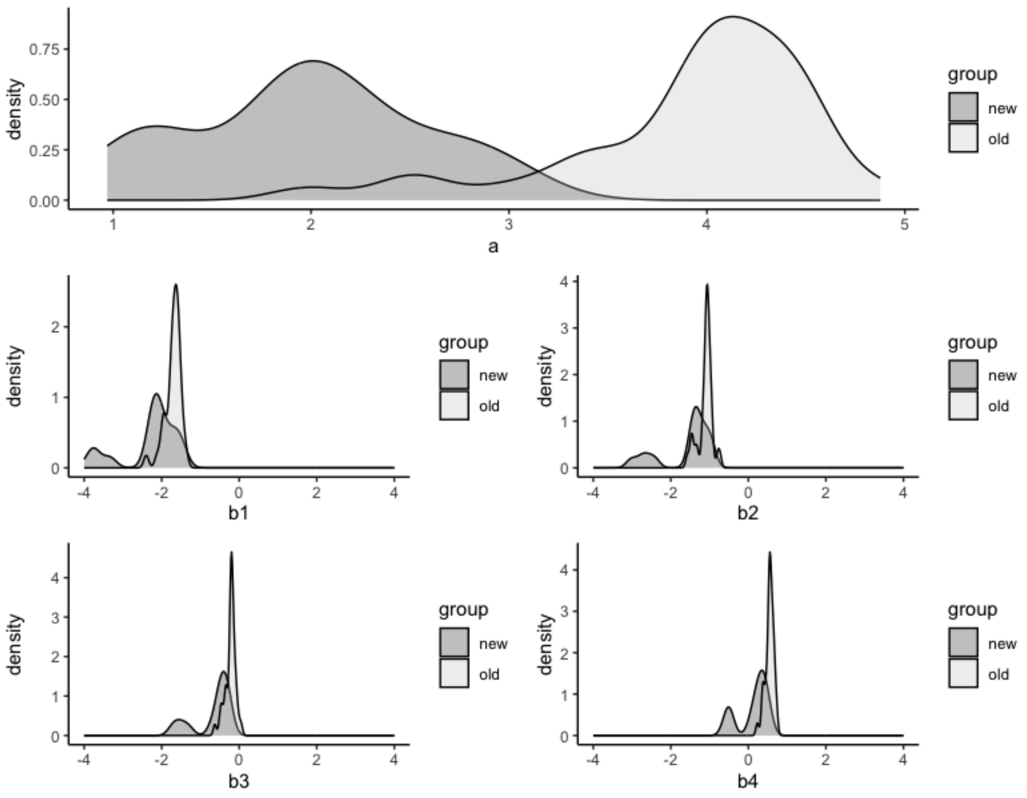

Figure S3. Bland-Altman plot Comparing Individual Theta Scores Between Old and New Item Bank

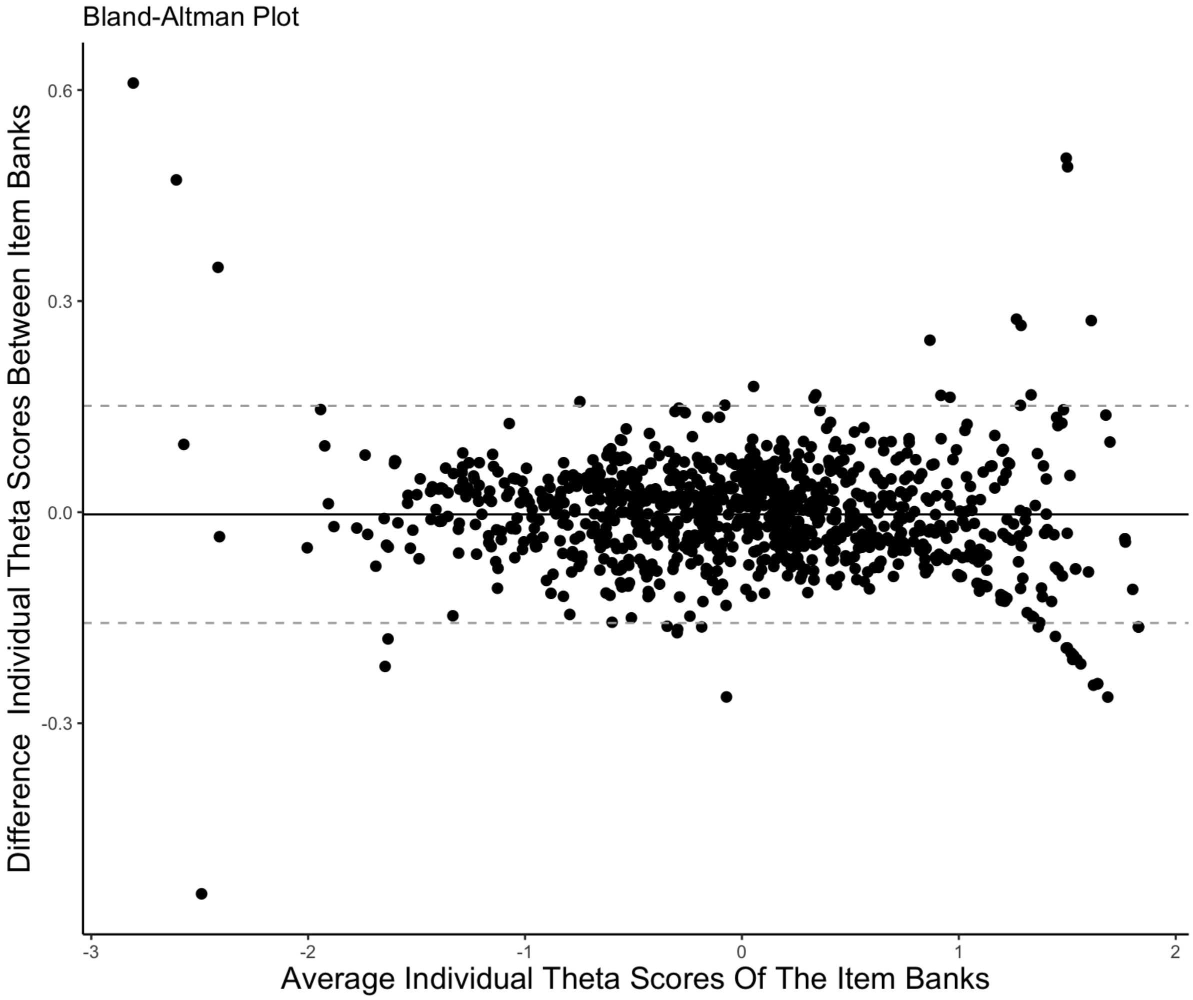

Note: A Bland-Altman plot describes the agreement between two measurements. The x-axis represents the average of the two measurements being compared. Here it shows the average θ score for each respondent on the old and new item bank. For example, if a respondent has a θ score of 1 on the old item bank and a θ score of 1.6 on the new item bank, the average would be 1.3. The y-axis represents the difference between these two measurements, in this example .6. Hence for this respondent a dot is plotted at x = 1.3 and y = .6. This is repeated for all respondents. In the Bland-Altman plot, a solid horizontal line represents the mean difference between the two measurements. This line serves as a reference for any systematic bias, indicating whether one measurement consistently tends to be higher or lower than the other. In our case, this line is centered around zero, suggesting that, in general, there is no significant difference in individual θ scores between the old and new item banks. However, the spread of data points on the y-axis showes a marked increase at the extreme ends

of the θ scores on the x-axis. This indicates that the lack of difference between the item banks is not consistent across all levels of θ, especially at the extreme values. The two dashed horizontal lines, positioned at a distance of ±1.96 standard deviations (SD) from the mean difference line, define the Limits of Agreement (LoA). These LoA defines the range in which 95% of the differences in θ between the old and new item banks lie. In our case, this range is approximately ± 0.15, which is relatively narrow. This suggests that, on the whole, the two measurements generally agree with each other. However, there are outliers at the more extreme levels of θ. In summary, the Bland-Altman plot indicates an overall good agreement between the old and new item banks, except for the extreme levels of θ.

Table S1. Mokken Item Scalability Coefficients

Item (Old)	Item H	se	Item (New)	Item H	se
RP1	0.560	0.017	PEXP_1	0.362	0.026
RP6	0.533	0.018	PEXP_2	0.561	0.019
SRPPER_CaPS1	0.555	0.018	PEXP_3	0.571	0.016
SRPPER01r1	0.545	0.019	PEXP_4	0.598	0.018
SRPPER02r1	0.578	0.016	PEXP_5	0.499	0.020
SRPPER03r1	0.558	0.018	PEXP_6	0.496	0.022
SRPPER04_CaPS	0.568	0.016	PEXP_7	0.443	0.022
SRPPER05_CaPS	0.571	0.018	PEXP_8	0.466	0.022
SRPPER06_CaPS	0.618	0.014	PEXP_9	0.524	0.021
SRPPER07_CaPS	0.605	0.015	PEXP_10	0.507	0.021
SRPPER08_CaPS	0.622	0.016	PEXP_11	0.367	0.026
SRPPER09_CaPS	0.632	0.014	PEXP_12	0.387	0.026
SRPPER11_CaPS	0.644	0.014	PEXP_13	0.568	0.017
SRPPER13_CaPS	0.592	0.017	PEXP_14	0.472	0.022
SRPPER14r1	0.633	0.015	PEXP_15	0.511	0.020
SRPPER15_CaPS	0.597	0.015	PEXP_16	0.306	0.028
SRPPER16r1	0.575	0.017	PEXP_17	0.463	0.020
SRPPER17r1	0.570	0.016			
SRPPER18_CaPS	0.631	0.014			
SRPPER20_CaPS	0.629	0.015			
SRPPER21_CaPS	0.642	0.015			
SRPPER22_CaPS	0.624	0.015			
SRPPER23_CaPS	0.625	0.015			
SRPPER26_CaPS	0.647	0.014			
SRPPER28r1	0.630	0.015			
SRPPER31_CaPS	0.586	0.018			
SRPPER35_CaPS	0.637	0.014			
SRPPER36_CaPS	0.646	0.014			
SRPPER37_CaPS	0.637	0.014			
SRPPER42r1	0.617	0.015			
SRPPER43r1	0.502	0.020			
SRPPER46_CaPS	0.652	0.014			
SRPPER47_CaPS	0.629	0.015			
SRPPER54_CaPS	0.645	0.014			
SRPPER55r1	0.560	0.018			
